# Sodium lactate improves renal microvascular thrombosis compared to sodium bicarbonate and 0.9% NaCl in a porcine model of endotoxic shock: an experimental randomized open label controlled study

**DOI:** 10.1186/s13613-018-0367-9

**Published:** 2018-02-14

**Authors:** Thibault Duburcq, Arthur Durand, Antoine Tournoys, Viviane Gnemmi, Valery Gmyr, François Pattou, Mercedes Jourdain, Fabienne Tamion, Emmanuel Besnier, Sebastien Préau, Erika Parmentier-Decrucq, Daniel Mathieu, Julien Poissy, Raphaël Favory

**Affiliations:** 1 Centre de Réanimation - Rue Emile Laine, CHU de Lille – Hôpital R Salengro, 59037 Lille Cedex, France; 2 INSERM U1190 Translational Research for Diabetes, Univ Lille, 59000 Lille, France; 3 European Genomic Institute for Diabetes, 59000 Lille, France; 4 Centre de Biologie Pathologie, CHU Lille, 59000 Lille, France; 5 Medical Intensive Care Unit, Rouen University Hospital, Rouen, France; 6 LIRIC Inserm U995 Glycation: From Inflammation to Aging, 59000 Lille, France

**Keywords:** Septic shock, Fluid resuscitation, Lactate infusion, Glomerular filtration rate, Disseminated intravascular coagulation, Renal histology

## Abstract

**Background:**

Sodium lactate seemed to improve fluid balance and avoid fluid overload. The objective of this study was to determine if these beneficial effects can be at least partly explained by an improvement in disseminated intravascular coagulation (DIC)-associated renal microvascular thrombosis.

**Methods:**

Ancillary work of an interventional randomized open label controlled experimental study. Fifteen female “Large White” pigs (2 months old) were challenged with intravenous infusion of *E. coli* endotoxin. Three groups of five animals were randomly assigned to receive different fluids: a treatment group received sodium lactate 11.2% (SL group); an isotonic control group received 0.9% NaCl (NC group); a hypertonic control group, with the same amount of osmoles and sodium than SL group, received sodium bicarbonate 8.4% (SB group). Glomerular filtration rate (GFR) markers, coagulation and inflammation parameters were measured over a 5-h period. Immediately after euthanasia, kidneys were withdrawn for histological study. Statistical analysis was performed with nonparametric tests and the Dunn correction for multiple comparisons. A *p* < 0.05 was considered significant.

**Results:**

The direct immunofluorescence study revealed that the percentage of capillary sections thrombosed in glomerulus were significantly lesser in SL group [5 (0–28) %] compared to NC [64 (43–79) %, *p* = 0.01] and SB [64 (43–79), *p* = 0.03] groups. Alterations in platelet count and fibrinogen level occurred earlier and were significantly more pronounced in both control groups compared to SL group (*p* < 0.05 at 210 and 300 min). The increase in thrombin–antithrombin complexes was significantly higher in NC [754 (367–945) μg/mL; *p* = 0.03] and SB [463 (249–592) μg/mL; *p* = 0.03] groups than in SL group [176 (37–265) μg/mL]. At the end of the experiment, creatinine clearance was significantly higher in SL group [55.46 (30.07–67.85) mL/min] compared to NC group [1.52 (0.17–27.67) mL/min, *p* = 0.03].

**Conclusions:**

In this study, we report that sodium lactate improves DIC-associated renal microvascular thrombosis and preserves GFR. These findings could at least partly explain the better fluid balance observed with sodium lactate infusion.

## Background

Sepsis, considered today as a syndrome of physiologic, pathologic and biochemical abnormalities induced by infection [1], is a major public health concern responsible for considerable morbidity and mortality [2]. Sepsis is frequently complicated by acute kidney injury, which is associated with higher risk of in-hospital mortality [3, 4], and by disseminated intravascular coagulation (DIC) due to a massive activation of the coagulation system [5, 6]. Many studies imply that DIC is an important mediator in both microvascular thrombosis, multiple organ failure syndrome development [7] and mortality in patients with serious infections [8].

Sepsis is also associated with deficit in effective blood volume. Large amounts of intravenous fluids are commonly used to increase cardiac output and improve peripheral blood flow [9]. First, mounting evidence suggests that resuscitation fluids contribute, in varying degrees, to clinically relevant renal [10, 11] and haemostatic disturbances, particularly if artificial colloids such as hydroxyethyl starch (HES) and gelatine or saline preparations are used. The undesirable consequences of using HES resulted in a strong recommendation [9] against the use of HES in resuscitation of patients with sepsis [12, 13]. Moreover, saline with the presence of supraphysiological concentrations of chloride may increase the incidence of acute kidney injury and the use of renal replacement therapy [14–16]. Secondly, aggressive use of large-volume intravenous fluids induces fluid overload which is associated with renal failure [17, 18] and leads to hemodilution, which in turn may exacerbate coagulopathy [19]. In order to reduce the volume of intravenous solutions, the concept of small volume resuscitation with hypertonic saline and/or hypertonic saline–HES or dextran has been widely studied during trauma resuscitation [20, 21]. The potential of these hypertonic fluids to modulate the coagulation cascade is less well known, as data are limited and contradictory [22]. Anyway, hypertonic solutions containing HES and/or saline could increase acute kidney injury in sepsis as far as isotonic fluids. Hence, in an attempt to avoid the detrimental effects of chloride anion and/or HES, the use of metabolized anions such as lactate could be more suitable. The use of lactate, as a resuscitation fluid-based energetic substrate, is an interesting alternative because this anion is well metabolized [23] even in poor hemodynamic conditions [24].

We previously observed that sodium lactate infusion enhanced fluid balance in pig endotoxic shock [25, 26]. This beneficial effect of sodium lactate could not be totally explained neither by its hyperosmolar or alkalizing effects [25] nor by its energy load or its effect on the chloride balance [26]. Finally, two additional mechanisms have been hypothesized: first, lactate infusion is better metabolized in poor hemodynamic conditions than glucose, and second, lactate could decrease proinflammatory response and/or improve endothelial barrier function. Interestingly, it is well known that proinflammatory response and endothelial dysfunction exacerbate the endotoxin-induced DIC [27, 28]. So, in order to better understand the beneficial effect of sodium lactate on fluid balance, we conducted an ancillary work focused on DIC and renal histology.

The main objective of the present study was to determine if sodium lactate improve DIC-associated renal microvascular thrombosis. The secondary objective was to explore the glomerular filtration rate (GFR).

## Methods

This is an ancillary work of a recent experimental study on the beneficial hemodynamic and metabolic effects of sodium lactate infusion in endotoxic shock [26]. The experimental protocol (CEEA No. 132012) received the approval of the Nord-Pas-de-Calais Animal Ethics Committee (Comité d’Ethique en Expérimentation Animale Nord-Pas-de-Calais; C2EA-75) and the French Ministry of Education and Research. Care and handling of the animals were in accordance with the experimental animal use guidelines of the French Ministry of Agriculture and Food.

### Animal preparation

For the experiment, animals were premedicated with intramuscular injection of ketamine (Kétalar^®^, Virbac, France, 2.5 mg/kg of body weight) and xylazine (Sédaxylan^®^, CEVA Santé Animale, France, 2.5 mg/kg of body weight). Then, we used isoflurane (AErrane^®^, Baxter, France) for the intubation process, and maintenance of anaesthesia was performed with a continuous infusion of midazolam (Hypnovel^®^, Roche, France, 1 mg/kg body weight/h) for the whole experiment. All animals were mechanically ventilated (Osiris 2^®^, Taema, France) with a tidal volume of 8 mL/kg, a positive end-expiratory pressure set at 4 cm H_2_O to limit cardiovascular effects, FiO_2_ 0.6 to prevent fatal hypoxaemia during the study, and respiratory rate 20–24 breaths/min only adjusted to maintain normocapnia (40–45 mmHg) at baseline. We chose to maintain ventilation similar in all animals during the experiment. No recruitment manoeuvres were done. Muscle relaxation was obtained by a continuous intravenous infusion of cisatracurium besylate (Nimbex^®^, Hospira, France, 2 mg/kg body weight/h). Analgesia was achieved by a subcutaneous injection of buprenorphine (Vetergesic^®^, Sogeval, France, 0.1 mg/kg body weight). After dissection of neck vessels, catheters were inserted in the right carotid artery for continuous blood pressure monitoring and blood sampling. To monitor urine output, a suprapubic urinary catheter was inserted.

### Study design

**Fig. 1:**
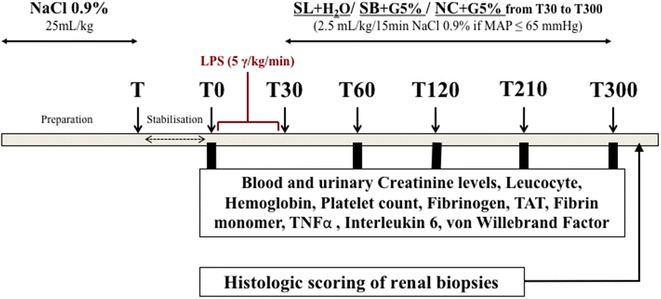
Study design. During preparation period, all animals received 25 mL/kg 0.9% NaCl to prevent hypovolemia. Measurements were taken over a 5-h period: at baseline (T0) and at 60 (T60), 120 (T120), 210 (T210) and 300 (T300) minutes. All animals were administered 5 μg/kg/min Escherichia coli lipopolysaccharide (LPS). The endotoxin was infused over a 30-min period intravenously. The SL group received 40.5 g lactate (3.61 kcal/g). NC and SB groups received an equivalent energy supply: 39 g glucose (3.75 kcal/g) as 780 mL 5% glucose solution from T30 to T300. To ensure the same fluid intake, the SL group received 780 mL sterile water for injection. If mean arterial pressure (MAP) felt below 65 mmHg, 2.5 mL/kg infusion of NaCl 0.9% was given as rescue therapy every 15 min. At the end of the study period, all animals were sacrificed with T61 administration. Immediately after euthanasia, renal biopsies were performed

Fifteen female “Large White” pigs (2 months old) were used in this study. The study was carried out as depicted in Fig. 1. During preparation period, animals received 25 mL/kg 0.9% NaCl to prevent hypovolemia. Measurements were taken over a 5-h period: at baseline after the stabilization period (T0) and at 60 (T60), 120 (T120), 210 (T210) and 300 (T300) minutes. All animals were administered 5 μg/kg/min Escherichia coli lipopolysaccharide (LPS) (serotype 055:B5; Sigma Chemical Co., St. Louis, MO, USA). The endotoxin was diluted in 50 ml of 0.9% NaCl and infused over a 30-min period intravenously. We studied three groups receiving 450 mL (from T30 to T300) of different fluids as follows: a treatment group (*n* = 5) receiving 11.2% hypertonic sodium lactate AP-HP^®^ (AGEPS, Paris, France) (**SL group**) containing 90 g (1000 mmol) of lactate and 23 g (1000 mmol) of sodium per litre and two control groups; one isotonic control group (*n* = 5) receiving 0.9% NaCl (**NC group**), and one hypertonic control group (*n* = 5) receiving 8.4% hypertonic sodium bicarbonate (**SB group**) containing 61 g (1000 mmol) of bicarbonate and 23 g (1000 mmol) of sodium per litre. Sodium bicarbonate provided the same amount of sodium (450 mmol) and osmoles (900 mosm), and the same alkalizing effect than sodium lactate [25]. The SL group received 40.5 g lactate (3.61 kcal/g). NC and SB groups received an equivalent energy supply: 39 g glucose (3.75 kcal/g) as 780 mL 5% glucose solution (Baxter SAS, Guyancourt, France) from T30 to T300. Finally, the SL group received 780 mL sterile water for injection (Baxter SAS, Guyancourt, France) in place of the 5% glucose solution to ensure the same fluid intake in the three groups. The only resuscitation endpoint was mean arterial pressure (MAP). If MAP felt below 65 mmHg, 2.5 mL/kg infusion of NaCl 0.9% was given as rescue therapy every 15 min. Bolus infusions were performed to maintain MAP above 65 mmHg as recommended by Sepsis Surviving Campaign [9]. At the end of the study period, all animals were sacrificed with T61 administration (T61, 0.3 mL/kg of body weight, Intervet International GmbH, Köln, Germany).

### Histological analysis

At the end of the experiment and immediately after euthanasia, kidneys were withdrawn for histological study. After macroscopic examination, a part of the samples from each kidney were fixed with acidified formal alcohol (AFA) and another part of the sample was frozen by liquid nitrogen and stored at − 80 °C. The samples fixed in AFA were embedded in paraffin and sectioned (3–4 μm width). After deparaffinization and rehydration, sections were stained with Masson’s trichrome, Periodic acid–Schiff (PAS) and hematoxylin eosin safran (HES) and evaluated in light microscopy. On frozen tissue, cryosections of 5 μm were cut on a cryostat and incubated 30 min with an antifibrinogen antibody directly conjugated with fluorescein for a direct immunofluorescence. The antibody was a polyclonal rabbit antibody, which recognized fibrinogen (ref. F0111, Dako SA, Trappes, France). We established two semiquantitative scores for histological abnormalities defined as: score (%) = (number of glomeruli damaged)/(number of glomeruli examined) and (number of capillary sections thrombosed)/(number of capillary sections examined) per damaged glomeruli. At least 50 glomeruli were observed in each sample. The pathologist was blinded to the groups examined.

### Biological methods

Leucocyte, haemoglobin and platelet counts were obtained on EDTA anticoagulated blood. For coagulation assays, blood (four parts) was collected in tubes containing 3.8% sodium citrate (one part). Fibrinogen levels were rapidly measured by standard procedures. Immunoassay methods were used to determine quantitative thrombin–antithrombin complexes (TAT) (Enzygnost^®^ TAT micro, Siemens, Munich, Germany). Fibrin monomer (Liatest FM^®^ Stago, Asnières, France) was performed by immunoturbidimetric assay. The quantitative determination of vWF antigen (Ag) was measured by turbidimetric assay (vWF Ag^®^ Reagent, Siemens, Nederland) (*n* = 70–100%).

TNFα and interleukin-6 (Il-6) were measured in serum. Plasma levels were detected by ELISA method with porcine anti-TNFα antibodies (Quantikine^®^ Porcine TNFα, R&D Systems, USA) and anti-Il-6 antibodies (Quantikine^®^ Porcine Il-6, R&D Systems, USA).

Blood and urinary creatinine levels (Cobas^®^ 8000 modular analyser, Roche Diagnostics, Switzerland) were measured at each time except T30. We computed creatinine clearance, a surrogate marker of GFR, with standard formula [creatinine clearance (CrCl) = (creatinine urinary concentration × rate of urine formation)/Creatinine plasma concentration]. Diuresis, a marker of both GFR and tubular function, was measured at each time except T30.

### Data analysis

We considered that the sample size of five animals per group would be sufficient to show a statistical difference if any based on a previous work on the same model [27]. Statistical analysis was performed with GraphPad Prism 6 software (San Diego, California). As the distribution was not normal (Shapiro–Wilk test), quantitative data were expressed using median and interquartile range. Considering the differences between groups for some parameters at baseline, values are expressed as a percentage of the first value. For multiple intergroup testing, we used Kruskal–Wallis test with Dunn’s multiple comparisons test and Mann–Whitney *U* test. Intragroup comparisons were realized by Friedman test with Dunn’s multiple comparisons test and Wilcoxon matched-pairs signed rank test. The two-tailed significance level was set at *p* < 0.05.

## Results

Median weight was similar in the three groups of animals: 22.5 (18.25–23.75) kg in NC group, 23 (21.75–23.5) kg in SB group and 23 (20.5–24) kg in SL group.

The endotoxin challenge resulted in hypodynamic shock with a decreased of cardiac index in all animals. As already described, the infusion of sodium lactate infusion enhanced hemodynamics with a limitation of fluid overload [26].

### Histological results

**Fig. 2:**
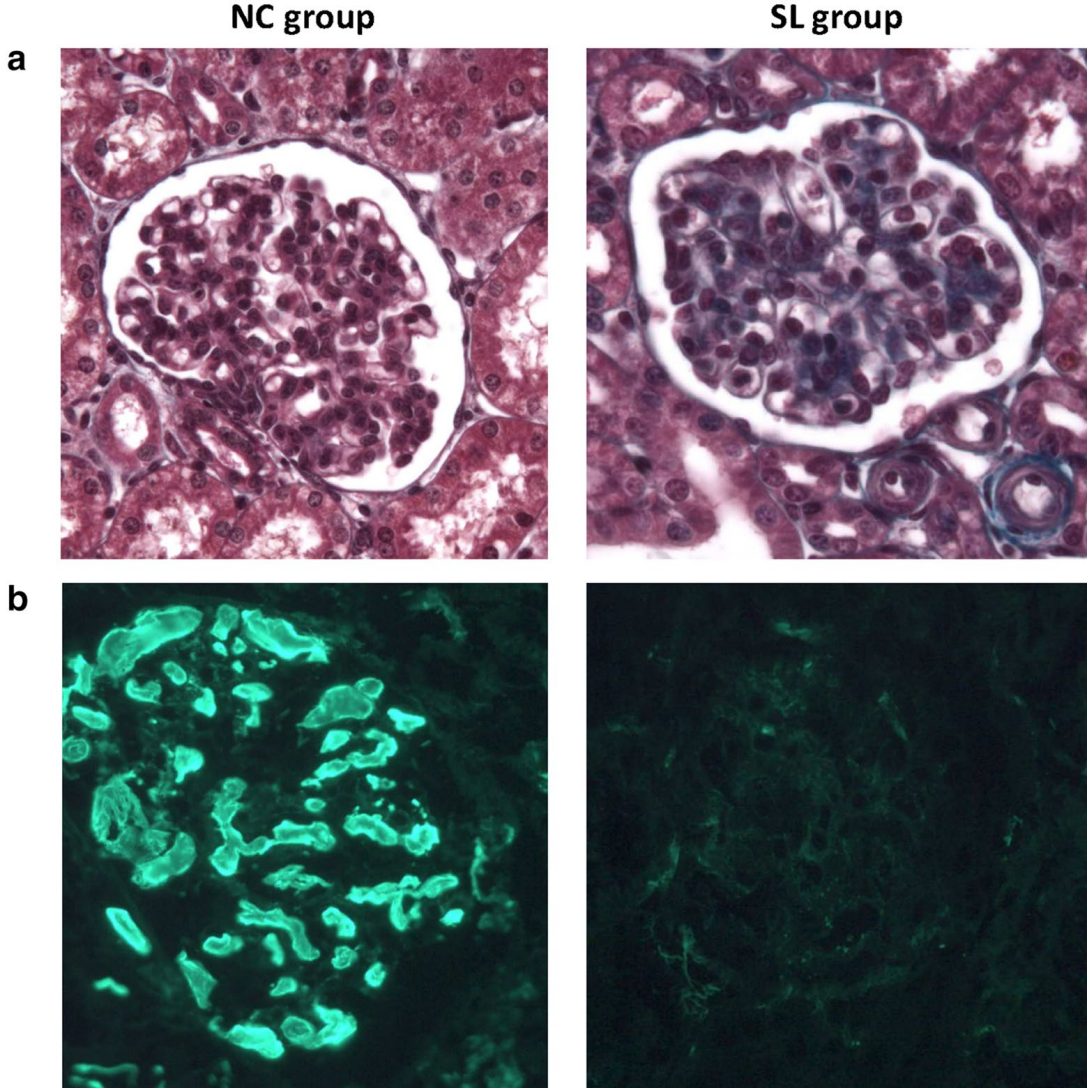
Histological comparison of NC and SL groups samples. Light microscopy (Masson’s trichrome, magnification ×400) (**a**) and immunofluorescence study with polyclonal antifibrinogen antibody (**b**) of a kidney section. In NC group sample, glomeruli showed signs of oedema uniformly, with glomerular capillary thrombosis well estimated by immunofluorescence study

Kidneys appeared macroscopically enlarged and swollen in the three groups. Glomeruli showed signs of oedema uniformly, and fibrin thrombi were mainly observed in glomerular capillaries (Fig. 2). Percentage of thrombosed glomeruli and percentage of thrombosed capillary in glomerulus were significantly higher in control groups compared to SL group (Table 1) and consistent with an increased amount of microthrombosis.

**Table 1:** Semiquantitative histological scores

Groups	Percentage of thrombosed glomeruli		Percentage of capillary sections thrombosed	
	Light microscopy	Immunofluorescence	Light microscopy	Immunofluorescence
NC	95 (42–100)	96 (54–100)	58 (31–69)	64 (43–79)
SB	96 (41–100)	94 (46–100)	57 (39–75)	68 (31–77)
SL	14 (0–43)	10 (0–49.20)	5 (0–32)	5 (0–28)
*p*				
NC versus SB	Ns	Ns	Ns	Ns
SL versus NC	*p* = 0.03	*p* = 0.03	*p* = 0.04	*p* = 0.01
SL versus SB	*p* = 0.03	*p* = 0.03	*p* = 0.02	*p* = 0.03

Percentage of thrombosed glomeruli (%) = (number of glomeruli damaged)/(number of glomeruli examined) and percentage of capillary sections thrombosed (%) = (number of capillary sections thrombosed)/(number of capillary sections examined) per damaged glomeruli. Results are expressed as median with interquartile ranges. Kruskal–Wallis test with Dunn’s multiple comparisons test and Mann–Whitney U test were used for intergroup comparisons.

### Coagulation and endothelial parameters

**Fig. 3:**
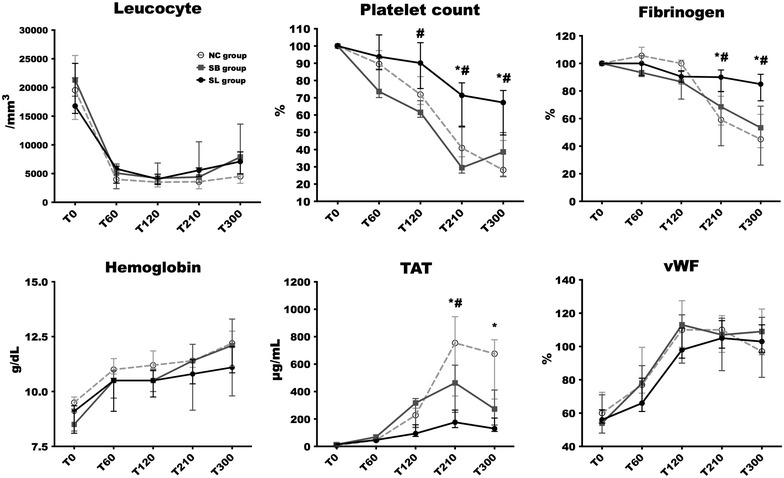
Changes in leucocyte, platelet count, fibrinogen, haemoglobin, TAT and vWF in the three groups. Considering the differences between groups for platelet count and fibrinogen at baseline, values are expressed as a percentage of the first value. Open circles and dotted line: NC group (*n* = 5); squares and grey line: SB group (*n* = 5); closed circles and black line: SL group (*n* = 5). Results are expressed as median with interquartile ranges. *p < 0.05, NC versus SL. ^#^*p* < 0.05, SB versus SL. ^&^*p* < 0.05, NC versus SB

Changes in leucocyte, platelet count, fibrinogen, haemoglobin, TAT and vWF in the three groups are illustrated in Fig. 3. As expected, we observed a dramatic procoagulant response. Alterations in platelet count and fibrinogen level occurred earlier and were significantly more pronounced in both control groups compared to SL group. Circulating platelets significantly declined at T300 in NC [28 (24–45) %; *p* = 0.03 compared to baseline] and SB [38 (24–50) %; *p* = 0.03 compared to baseline] groups, while in SL group, the decrease in the platelet count was less important [67 (48–74) %; *p* = 0.06 compared to baseline]. In the same way, the activation of the coagulation cascade was illustrated by a decrease in circulating fibrinogen. Fibrinogen level significantly declined at T300 in NC [45 (39–63) %; *p* = 0.03 compared to baseline] and SB [53 (26–69) %; *p* = 0.03 compared to baseline] groups, while it remained stable in SL group [85 (73–92) %; *p* = 0.06 compared to baseline]. The increase in fibrin monomer started earlier and was significantly higher at T120 in NC [104 (82–175) μg/mL; *p* = 0.01] and SB groups [161 (69–200) μg/mL; *p* = 0.03] compared to SL group [28 (19–74) μg/mL]. Unfortunately, we could not interpret the results at 210 and 300 min in the three groups because some values were over 200 μg/mL, the upper limit of measurement, despite dilutions (data not shown). Thrombin–antithrombin complex (TAT) concentrations started to increase at T60 to achieve a maximum level at T210. The increase in TAT complexes was earlier and significantly higher in NC [754 (367–945) μg/mL; *p* = 0.03] and SB [463 (249–592) μg/mL; *p* = 0.03] groups than in SL group [176 (37–265) μg/mL]. Von Willebrand factor (vWF) increased in all animals without any significant difference between groups.

### Inflammation parameters

**Fig. 4:**
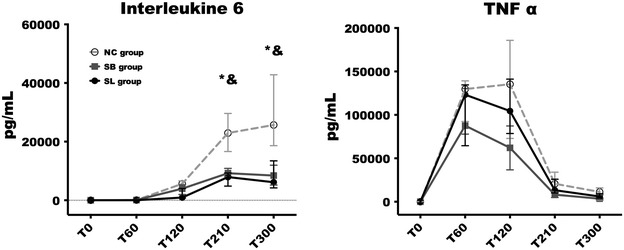
Changes in interleukin-6 and TNFα in the three groups. Open circles and dotted line: NC group (*n* = 5); squares and grey line: SB group (*n* = 5); closed circles and black line: SL group (*n* = 5). Results are expressed as median with interquartile ranges. *p < 0.05, NC versus SL. ^#^*p* < 0.05, SB versus SL. ^&^*p* < 0.05, NC versus SB

Changes in interleukin-6 and TNFα in the three groups are illustrated in Fig. 4. We observed a same evolution of TNFα levels in the three groups without any significant differences. TNFα increased rapidly, peaked at T120 in both hypertonic groups and at T210 in NC group, and subsequently decreased until the end of the experiment without returning to baseline levels. Il-6 was significantly higher in NC group [22,938 (16,619–29,613) pg/mL at T210 and 25,687 (18,617–42,792) pg/mL at T300] compared to SL group [7904 (4838–9310) pg/mL at T210, *p* = 0.02 and 6148 (4216–13,445) at T300, *p* = 0.03] and SB group [9234 (8108–10,869) pg/mL at T210, *p* = 0.02 and 8433 (5174–11,961) at T300, *p* = 0.02]. No significant differences were seen on Il-6 evolution between SB and SL groups at any time.

### Glomerular filtration rate (GFR) markers

**Fig. 5:**
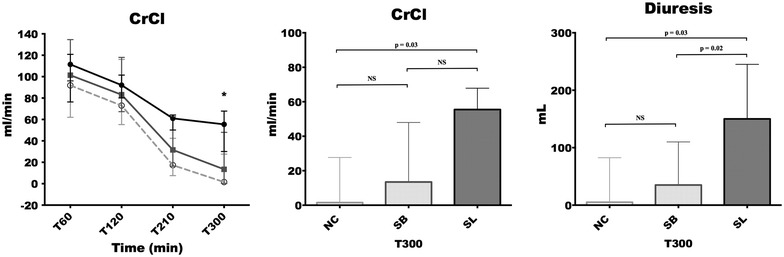
Creatinine clearance (CrCl) and diuresis in the three groups. Open circles and dotted line: NC group (*n* = 5); squares and grey line: SB group (*n* = 5); closed circles and black line: SL group (*n* = 5). Results are expressed as median with interquartile ranges. *p < 0.05, NC versus SL. ^#^*p* < 0.05, SB versus SL. ^&^*p* < 0.05, NC versus SB

Creatinine clearance (CrCl) and diuresis in the three groups are illustrated in Fig. 5. At the end of the experiment (between T210 and T300), diuresis was significantly higher in SL group [150 (125–245) mL] compared to NC [5 (2.5–82.5) mL, *p* = 0.03] and SB groups [35 (1–110), *p* = 0.02]. Creatinine clearance was higher in SL group [55.46 (30.07–67.85) mL/min] compared to NC [1.52 (0.17–27.67) mL/min, *p* = 0.03] and SB groups [13.46 (0.31–47.99) mL/min, *p* = 0.09].

## Discussion

In the present study, we compare hypertonic sodium lactate with two different therapeutic regimens; (1) a standard fluid therapy with isotonic crystalloids (0.9% NaCl, the most commonly used crystalloid). Although comparing hypertonic with isotonic formulations could appear misleading, it seemed necessary to have a control group corresponding to the usual clinical practice. (2) a non-conventional hypertonic fluid therapy. Due to an acidifying effect on pH and an elevated chloride concentration, hypertonic saline was not close enough to hypertonic sodium lactate. Conversely, sodium bicarbonate provided the same alkalizing effect and the same amount of sodium and osmoles than sodium lactate. Thereby, this comparison allows extracting clear conclusions and physiological assumptions. We report here that sodium lactate infusion improves DIC-associated renal microvascular thrombosis. The decrease in renal microvascular thrombosis in SL group could be at least partly explained by the delayed and attenuated procoagulant response (sodium lactate infusion resulted in a significant smaller decrease in platelets and fibrinogen concentrations and a significant smaller increase in plasma levels of TAT). It is known that glomerular thrombosis and vascular thrombosis due to the activation of inflammation and coagulation pathway contribute to the occurrence of acute renal failure in sepsis [5, 29]. Indeed, glomerular thrombosis and microvascular fibrin thrombosis compromise glomerular capillary flow, leading to focal ischaemia and necrosis, which is considered to be the main pathogenesis of LPS-induced acute renal failure [29, 30].

We first hypothesized that sodium lactate infusion may reduce the endothelial dysfunction and therefore restrict the coagulation cascade. In fact, it is known that endothelial dysfunction precedes derangement of platelet function or coagulation parameters and drives a pre-DIC-associated microvascular thrombosis in endotoxemia [28]. In paediatric severe Dengue infection, hypertonic sodium lactate induced a partial recovery from endothelial dysfunction, as indicated by a significant decrease in sVCAM-1 [31]. Moreover, lactate as a metabolizable anion may lead to chloride egress from endothelial cells, causing reduction in swelling and improvement in barrier function [32]. In our model, we already observed that sodium lactate infusion seemed to reduce capillary leakage [26]. In the same way, we observed a non-significant lesser haemoconcentration with lactate infusion in the present study. However, the evolution of von Willebrand factor, a marker of endothelial dysfunction, was not different between groups. Finally, further investigations focused on endothelial function are warranted to explore the sodium lactate impact on capillary leak.

An other explanation of the beneficial effect of sodium lactate on DIC could be an anti-inflammatory effect. It is known that excessive inflammatory mediators play a central role in the development of endotoxin-induced DIC. TNFα plays an important part in the early activation of the haemostatic mechanism and in the pathogenesis of DIC [33]. Indeed, a TNFα inhibitor can act as a protective drug in lipopolysaccharide-induced DIC in a dose-dependent manner [34]. Then, it is known that plasma IL-6 is higher in patients with DIC than in those without DIC. Some data suggest that increases in IL-6 might give rise to hypercoagulable and hypofibrinolytic states. IL-6 could be a cause of DIC and be related to prognosis and organ failure [35]. At last, immunoglobulin, in LPS-induced DIC model, could significantly decreased plasma levels of TNFα and IL-6 and improved haemostatic abnormality [36]. Experimental studies of sepsis showed beneficial effects of hyperosmolar solutions modulating inflammatory response, as for instance the expression and release of cytokines TNFα and IL-6 [37–39]. The use of hypertonic saline solution has also demonstrated potential anti-inflammatory effects related to neutrophil activation [40]. Hypertonic solution acts on polymorphonuclear A2 adenosine receptors and causes a feedback mechanism that stimulates cAMP and PKA release, thus blocking neutrophil activation [41, 42]. Nevertheless, the therapeutic window for a beneficial effect of fluid resuscitation with hypertonic fluid seemed to be very narrow [43] and may be related to leukocyte activation at the time of fluid use [44]. Our study confirms the beneficial impact of hypertonic solutions on the IL-6 release but not on TNFα. These results could explain part of the beneficial impact of sodium lactate infusion compared to saline. However, it does not elucidate the difference on coagulation status between SL and SB groups. In this way, another explanation could be that lactate by itself has important other anti-inflammatory properties. Binding of lactate on a specific membrane receptor (the plasma membrane GPR81) recruits the intracellular adaptor molecule ARRB2 to the receptor with subsequent inhibition of the NLRP3 inflammasome leading to a reduction in the Il-1β-mediated proinflammatory response [45]. Systemic LPS administration induced high levels of proinflammatory cytokines Il-1β, which contribute to the increased leukocyte–endothelium interaction and promote coagulation cascade [46].

Pigs were chosen as a clinically relevant species, resembling to humans in coagulation reactions [47]. Nevertheless, our model presents some limits. The length of evaluation is short, only 5 h. Bolus injection of endotoxin induces initial characteristics of human sepsis such as activation of innate immune system and rises in TNFα. Its short-term effects on the inflammatory cascade and the lack of an active nidus do not allow to study the compensatory anti-inflammatory phase often leading to immunosuppression. Nevertheless, endotoxin challenge is still a way to explore the very beginning of sepsis and the better resuscitation fluid strategy in this initial phase. Endotoxic model is not a model of hyperdynamic septic shock. Our model was a hypodynamic shock with a pronounced pulmonary vascular response. However, it reproduces some main alterations of inflammatory states, e.g. macrocirculatory and microcirculatory dysfunctions, coagulopathy, organs failure. We used the intravenous route for sepsis induction while patients are often infected by *natural route*. The temporal evolution of the aggression was imposed when the pathological process of patients following an individual natural progression. The endotoxin challenge is responsible for a more explosive proinflammatory response than in a septic shock. These limits, without questioning the validity of our pathophysiological model, could impair the comparability with DIC observed in human septic shock.

In this study, we also reported that sodium lactate preserves GFR. This finding could be at least partly explained by the slightest renal microvascular thrombosis but also by the hemodynamics and microcirculation improvements previously described [25, 26]. Two limits must be reported; (1) GFR do not accurately assess kidney function. Moreover, there is a dependence between diuresis and CrCl calculation which might have participated to the improvement in CrCl. (2) The mean arterial target threshold of 65 mmHg was not reached for two animals in isotonic saline group and one animal in sodium bicarbonate group. This may have impacted negatively the renal perfusion pressure during the resuscitation phase independently from the type of fluid resuscitation. Nevertheless, low blood pressure (< 65 mmHg) occurred only at the end of the experiment, and we already found severe oligo-anuria in these animals before mean arterial pressure falls below 65 mmHg.

## Conclusions

In conclusion, sodium lactate improves DIC-associated renal microvascular thrombosis and preserves GFR in our model of endotoxic shock. In the same way as hemodynamics improvement previously observed, these findings could at least partly explain the preservation of fluid balance with sodium lactate.

In our model, the beneficial effect of sodium lactate on DIC-associated renal microvascular thrombosis could be related to an anti-inflammatory effect (e.g. blockage of the NLRP3 inflammasome). Further investigations are warranted to explain the underlying mechanisms and to assess the potential clinical benefits of sodium lactate resuscitation in human sepsis.

## Abbreviations

SL: Sodium lactate
SB: Sodium bicarbonate
NC: NaCl 0.9%
MAP: Mean arterial pressure
TAT: Thrombin–antithrombin complex
vWF: von Willebrand factor
TNFα: Tumour necrosis factor α
Il-6: Interleukin 6
Il-1β: Interleukin 1β
*E. coli*: *Escherichia coli*
LPS: Lipopolysaccharide
AKI: Acute kidney injury
CrCl: Creatinin clearance
GFR: Glomerular filtration rate

## References

[1] Singer M, Deutschman CS, Seymour CW, Shankar-Hari M, Annane D, Bauer M (2016). The third international consensus definitions for sepsis and septic shock (sepsis-3). JAMA.

[2] Fleischmann C, Scherag A, Adhikari NKJ, Hartog CS, Tsaganos T, Schlattmann P (2016). Assessment of global incidence and mortality of hospital-treated sepsis. Current estimates and limitations. Am J Respir Crit Care Med.

[3] Bellomo R, Kellum JA, Ronco C, Wald R, Martensson J, Maiden M (2017). Acute kidney injury in sepsis. Intensive Care Med.

[4] Bagshaw SM, Uchino S, Bellomo R, Morimatsu H, Morgera S, Schetz M (2007). Septic acute kidney injury in critically ill patients: clinical characteristics and outcomes. Clin J Am Soc Nephrol.

[5] Levi M, Ten Cate H (1999). Disseminated intravascular coagulation. N Engl J Med.

[6] Bakhtiari K, Meijers JCM, de Jonge E, Levi M (2004). Prospective validation of the International Society of Thrombosis and Haemostasis scoring system for disseminated intravascular coagulation. Crit Care Med.

[7] Gando S (2010). Microvascular thrombosis and multiple organ dysfunction syndrome. Crit Care Med.

[8] Angstwurm MWA, Dempfle C-E, Spannagl M (2006). New disseminated intravascular coagulation score: a useful tool to predict mortality in comparison with Acute Physiology and Chronic Health Evaluation II and Logistic Organ Dysfunction scores. Crit Care Med.

[9] Rhodes A, Evans LE, Alhazzani W, Levy MM, Antonelli M, Ferrer R (2017). Surviving sepsis campaign: international guidelines for management of sepsis and septic shock: 2016. Intensive Care Med.

[10] Mårtensson J, Bellomo R (2017). Does fluid management affect the occurrence of acute kidney injury?. Curr Opin Anaesthesiol.

[11] Severs D, Hoorn EJ, Rookmaaker MB (2015). A critical appraisal of intravenous fluids: from the physiological basis to clinical evidence. Nephrol Dial Transplant Off Publ Eur Dial Transpl Assoc Eur Ren Assoc.

[12] Rochwerg B, Alhazzani W, Sindi A, Heels-Ansdell D, Thabane L, Fox-Robichaud A (2014). Fluid resuscitation in sepsis: a systematic review and network meta-analysis. Ann Intern Med.

[13] Haase N, Perner A, Hennings LI, Siegemund M, Lauridsen B, Wetterslev M (2013). Hydroxyethyl starch 130/0.38–0.45 versus crystalloid or albumin in patients with sepsis: systematic review with meta-analysis and trial sequential analysis. BMJ.

[14] Yunos NM, Bellomo R, Hegarty C, Story D, Ho L, Bailey M (2012). Association between a chloride-liberal vs chloride-restrictive intravenous fluid administration strategy and kidney injury in critically ill adults. JAMA.

[15.] Semler MW, Wanderer JP, Ehrenfeld JM, Stollings JL, Self WH, Siew ED (2017). Balanced crystalloids versus saline in the intensive care unit: the SALT randomized trial. Am J Respir Crit Care Med.

[16] Krajewski ML, Raghunathan K, Paluszkiewicz SM, Schermer CR, Shaw AD (2015). Meta-analysis of high- versus low-chloride content in perioperative and critical care fluid resuscitation. Br J Surg.

[17] Prowle JR, Kirwan CJ, Bellomo R (2014). Fluid management for the prevention and attenuation of acute kidney injury. Nat Rev Nephrol.

[18] Ostermann M, Straaten HMO, Forni LG (2015). Fluid overload and acute kidney injury: cause or consequence?. Crit Care.

[19] Paydar S, Bazrafkan H, Golestani N, Roozbeh J, Akrami A, Moradi AM (2014). Effects of intravenous fluid therapy on clinical and biochemical parameters of trauma patients. Emerg Tehran Iran.

[20] Feinman M, Cotton BA, Haut ER (2014). Optimal fluid resuscitation in trauma: type, timing, and total. Curr Opin Crit Care.

[21] Gantner D, Moore EM, Cooper DJ (2014). Intravenous fluids in traumatic brain injury: what’s the solution?. Curr Opin Crit Care.

[22] Kaczynski J, Wilczynska M, Hilton J, Fligelstone L (2013). Impact of crystalloids and colloids on coagulation cascade during trauma resuscitation-a literature review. Emerg Med Health Care.

[23] Chioléro R, Schneiter P, Cayeux C, Temler E, Jéquier E, Schindler C (1996). Metabolic and respiratory effects of sodium lactate during short iv nutrition in critically ill patients. JPEN J Parenter Enteral Nutr.

[24] Chioléro RL, Revelly JP, Leverve X, Gersbach P, Cayeux MC, Berger MM (2000). Effects of cardiogenic shock on lactate and glucose metabolism after heart surgery. Crit Care Med.

[25] Duburcq T, Favory R, Mathieu D, Hubert T, Mangalaboyi J, Gmyr V (2014). Hypertonic sodium lactate improves fluid balance and hemodynamics in porcine endotoxic shock. Crit Care.

[26] Duburcq T, Durand A, Dessein A-F, Vamecq J, Vienne J-C, Dobbelaere D (2017). Comparison of fluid balance and hemodynamic and metabolic effects of sodium lactate versus sodium bicarbonate versus 0.9% NaCl in porcine endotoxic shock: a randomized, open-label, controlled study. Crit Care.

[27] Duburcq T, Tournoys A, Gnemmi V, Hubert T, Gmyr V, Pattou F (2015). Impact of obesity on endotoxin-induced disseminated intravascular coagulation. Shock.

[28] De Ceunynck KEP, Higgins SJ, Chaudhry SA, Parikh S, Flaumenhaft RC (2016). Dysfunctional endothelium drives a Pre-DIC state in endotoxemia. Blood.

[29] Schrier RW, Wang W (2004). Acute renal failure and sepsis. N Engl J Med.

[30] Hertig A, Rondeau E (2004). Role of the coagulation/fibrinolysis system in fibrin-associated glomerular injury. J Am Soc Nephrol JASN.

[31] Somasetia DH, Setiati TE, Sjahrodji AM, Idjradinata PS, Setiabudi D, Roth H (2014). Early resuscitation of Dengue Shock Syndrome in children with hyperosmolar sodium-lactate: a randomized single blind clinical trial of efficacy and safety. Crit Care.

[32] Hoffmann EK, Lambert IH, Pedersen SF (2009). Physiology of cell volume regulation in vertebrates. Physiol Rev.

[33] van der Poll T, Büller HR, ten Cate H, Wortel CH, Bauer KA, van Deventer SJ (1990). Activation of coagulation after administration of tumor necrosis factor to normal subjects. N Engl J Med.

[34] Yamamoto N, Sakai F, Yamazaki H, Nakahara K, Okuhara M (1996). Effect of FR167653, a cytokine suppressive agent, on endotoxin-induced disseminated intravascular coagulation. Eur J Pharmacol.

[35] Hoppensteadt D, Tsuruta K, Hirman J, Kaul I, Osawa Y, Fareed J (2015). Dysregulation of inflammatory and hemostatic markers in sepsis and suspected disseminated intravascular coagulation. Clin Appl Thromb Off J Int Acad Clin Appl Thromb.

[36] Asakura H, Takahashi Y, Kubo A, Ontachi Y, Hayashi T, Omote M (2006). Immunoglobulin preparations attenuate organ dysfunction and hemostatic abnormality by suppressing the production of cytokines in lipopolysaccharide-induced disseminated intravascular coagulation in rats. Crit Care Med.

[37] Theobaldo MC, Llimona F, Petroni RC, Rios ECS, Velasco IT, Soriano FG (2013). Hypertonic saline solution drives neutrophil from bystander organ to infectious site in polymicrobial sepsis: a cecal ligation and puncture model. PLoS ONE.

[38] Theobaldo MC, Barbeiro HV, Barbeiro DF, Petroni R, Soriano FG (2012). Hypertonic saline solution reduces the inflammatory response in endotoxemic rats. Clin Sao Paulo Braz.

[39] Coelho AMM, Jukemura J, Sampietre SN, Martins JO, Molan NAT, Patzina RA (2010). Mechanisms of the beneficial effect of hypertonic saline solution in acute pancreatitis. Shock.

[40] Angle N, Hoyt DB, Cabello-Passini R, Herdon-Remelius C, Loomis W, Junger WG (1998). Hypertonic saline resuscitation reduces neutrophil margination by suppressing neutrophil L selectin expression. J Trauma.

[41] Pascual JL, Khwaja KA, Ferri LE, Giannias B, Evans DC, Razek T (2003). Hypertonic saline resuscitation attenuates neutrophil lung sequestration and transmigration by diminishing leukocyte-endothelial interactions in a two-hit model of hemorrhagic shock and infection. J Trauma.

[42] Inoue Y, Tanaka H, Sumi Y, Woehrle T, Chen Y, Hirsh MI (2011). A3 adenosine receptor inhibition improves the efficacy of hypertonic saline resuscitation. Shock.

[43] Petroni RC, Biselli PJC, de Lima TM, Velasco IT, Soriano FG (2015). Impact of time on fluid resuscitation with hypertonic saline (NaCl 7.5%) in rats with LPS-induced acute lung injury. Shock.

[44] Ciesla DJ, Moore EE, Zallen G, Biffl WL, Silliman CC (2000). Hypertonic saline attenuation of polymorphonuclear neutrophil cytotoxicity: timing is everything. J Trauma.

[45] Hoque R, Farooq A, Ghani A, Gorelick F, Mehal WZ (2014). Lactate reduces liver and pancreatic injury in Toll-like receptor- and inflammasome-mediated inflammation via GPR81-mediated suppression of innate immunity. Gastroenterology.

[46] Zhou J, Schmidt M, Johnston B, Wilfart F, Whynot S, Hung O (2011). Experimental endotoxemia induces leukocyte adherence and plasma extravasation within the rat pial microcirculation. Physiol Res.

[47] Hildebrand F, Andruszkow H, Huber-Lang M, Pape H-C, van Griensven M (2013). Combined hemorrhage/trauma models in pigs-current state and future perspectives. Shock.

